# Epidemiology and clinical features of Rotavirus infection among children in Rawalpindi, Pakistan

**DOI:** 10.1371/journal.pone.0324037

**Published:** 2025-05-20

**Authors:** Kiren Mustafa, Rani Faryal, Muhammad Masroor Alam, Suleman Rana, Massab Umair, Tawaf Ali Shah

**Affiliations:** 1 Department of Virology, National Institute of Health, Chak Shahzad, Islamabad, Pakistan; 2 Department of Microbiology, Faculty of Biological Sciences, Quaid-I-Azam University, Islamabad, Pakistan; Imam Abdulrahman Bin Faisal University, SAUDI ARABIA

## Abstract

Group A rotavirus (RVA) associated gastroenteritis is a major cause of infantile morbidity and mortality, globally. Pakistan had the highest rates of gastroenteritis among kids, every year. Our study aimed to assess the RVA disease burden and circulating genotypes in Rawalpindi, before the vaccine’s introduction in Pakistan. Stool samples were collected from children < 5 years of age admitted at Benazir Bhutto Hospital, Rawalpindi, from November 2014 to May 2015. Of the 300 stool samples, 47% of children were found positive for RVA antigen on ELISA, with the highest prevalence (52%) in infants less than 7 months of age. Rotavirus positive cases through real-time PCR were 65.5%. Fever and diarrhea were significantly related to RVA infection when compared to RVA-negative cases (*P* = 0.02). It is the first report on an upsurge of G12P[6] (17.24%) along with the rise of previously declining G3 in the current epidemiological area. The other prevalent types were G1P[8] (12.07%), G1P[4] (6.90%), G1P[6] (5.17%), G3P[6] (5.17%), followed by G2P[6], G3P[4], G9P[4], and G12P[8] each found with a prevalence of (3.45%). This study reports G3P[4], G3P[6] and G12P[4] for the first time in Pakistan. Mixed genotype infections were found in 21% of cases. G12P[6], which was the predominant genotype in this study, was found to be significantly associated with fever (*P* = 0.03). This study provides valuable data on the significantly highest prevalence of RVA-associated gastroenteritis in kids of Rawalpindi, Pakistan, and elucidates the vast diversity of circulating RVA genotypes. The reported disease burden, genotypes, and clinical symptoms would enable public health dealers to cope with the severity of the disease. It also provides an evolutionary trend of changing genotypes in the country.

## Introduction

Rotaviruses are the principal etiologic agent of acute gastroenteritis in the infantile population, accounting for 453,000 mortalities worldwide, annually [1,2]. Altogether, the poorly developed countries reported 50% of deaths among infants due to rotavirus, Pakistan alone accounted for > 60% of mortalities per 1 million total RVA gastroenteritis cases in kids [3]. Rotaviruses are double-stranded RNA viruses within the Reoviridae family, that contain seven serogroups based on antigens [4]. Group A rotavirus (RVA) is a principal aetiologic agent of ailment, it is a non-enveloped virus consisting of 11 dsRNA gene segments wrapped in trilaminar icosahedral capsid [5]. These segments encode for structural (VP1–4, VP6, VP7) and non-structural proteins (NSP1-NSP6) [6]. The outer layer of the capsid consists of two proteins, the glycoprotein (VP7) and a protease-sensitive spike protein (VP4), which are the basis of a binary system of RVA classification into G and P genotypes, respectively. The middle layer (VP6) and core protein VP2 enclose VP1 and VP3 [7]. The subgroups (A-H) are recognized based on immunoreactivity of VP6 and phylogenetic analysis [5,8].

G1P[8], G2P[4], G3P[8], G4P[8], and G9P[8] have been the most prevalent RVA strains in the world [9]. Based on molecular techniques, twenty-seven G and 37 P genotypes have been found in humans and animal species, worldwide [10]. In Pakistan, a cross-sectional study reported the highest frequency of G1P[8] in 2006–2008 [11]. G9 and G12 have been reported to rise in post-vaccination time in 2019 [12]. Another report declared the rise of G9P[4] in vaccinated kids in Pakistan during 2018–2020 [8]. New genotypes are emerging due to point mutations, interspecies transmission, and genetic re-assortment of gene segments among progeny RVA strains [3,13]. However, the prevalence of new genotypes can be challenging for the efficacy of the introduced vaccine.

Several studies have been carried out in many countries which provided important baseline data on epidemiological and clinical aspects of RVA [14–17]. Pakistan is among those countries in Asia where the infant mortality rate (> 100 deaths per 100,000 children per year) due to rotavirus is highest [3,18]. Still, little data is available on the clinical aspects of rotavirus disease and circulating genotypes from Pakistan [3,18^19^–^20^]. Our study not only analyzed the epidemiological features of RVA, but also the clinical peculiarity of the disease as well, and possible association of disease severity with the circulating genotypes.

As economically Pakistan is facing a lot of challenges, along with dealing with viral infections. WHO recommended in 2009 the introduction of the Rotavirus vaccine in National immunization programs in Pakistan and other Asian countries with the highest disease burden. Pakistan’s Ministry of Health initiated a hospital-based surveillance program to test the stool samples every year from children with gastroenteritis at central district hospitals in 3 large cities; Rawalpindi, Karachi, and Lahore, to overview the disease burden in major cities of Pakistan [21]. Our study being a part of the National rotavirus surveillance program fills the gap in the literature by providing rotavirus disease burden and circulating genotypes from RGH from 2014 to 2015.

Although the Rotarix vaccine was introduced in the EPI program of Pakistan in 2018, however, the vaccine has not proved as effective as we see in high-income countries [3]. Rotarix vaccine is based on attenuated monovalent strain G1P[8], later on, that was found more than 80% effective in high-income countries, nearly 70% effective in middle-income countries, and nearly 40% effective in low-income countries [12]. A study conducted by researchers at Agha Khan University (2017–2023) reported that the introduced vaccine has proved nearly 30% effective in Pakistan, which is far lower than the 80% efficiency observed in wealthier countries [18,22]. The reason behind this could be due to the high rotavirus disease burden and different predominant genotypes, so the introduced vaccine may need certain amendments based on reported epidemiological/clinical data in pre-vaccine time zones, at a large scale in the country. The current study is one of the reports, on the layout of circulating RVA genotypes, that would provide an evolutionary trend of the predominant genotypes and severity of the disease in Rawalpindi, Pakistan, in a specific time. It will also help to ascertain whether the genotypes and severity of clinical symptoms have any mutual relation among the infected kids of Pakistan.

## Materials and methods

### Ethical approval

The collection of stool samples from subjects under 5 years of age, from hospitals under the NIH routine Rotavirus surveillance program in Pakistan has been reviewed and approved by the Department of Microbiology, Quaid-I-Azam University, Islamabad. The collection of stool samples and relevant clinical data was conducted by the virology laboratory under the ethical guidelines for written/informed consent from parents/guardians, by the National Institute of Health, Islamabad-Pakistan. The stool samples and relevant clinical data/information were used solely for research purposes, under the NIH surveillance program to screen the prevalence of Rotavirus/genotypes in the respective population.

### Sample collection and analysis

Three hundred fecal/stool samples were collected in vials from children under 5 years of age, admitted to Rawalpindi General Hospital (RGH), as per WHO case standard definition, from children presenting watery non-bloody diarrhea. The nosocomial diarrhea was excluded. The sampling was done with informed consent from Parents/ Guardians of Patients. After collection, the samples were transported in cold boxes to the serology laboratory, Department of Virology, NIH Islamabad, Pakistan. Where initially they were stored at (-20° C) and then analyzed immediately within a week. The demographic, clinical, and stool data was collected from patients on standard data sheets with prior verbal consent from the patient’s guardian.

### Sample processing

#### Dilution of samples.

Fecal suspensions were prepared using sterile 1.5 ml microcentrifuge tubes in biosafety cabinets to avoid contamination. The samples were diluted in Phosphate buffered saline (PBS) to 10% fecal suspension by addition of 0.1g of solid fecal sample or 100 µl of liquid sample in 1 ml of PBS by using micropipettes with sterile tips. Before proceeding each microcentrifuge tube was labeled properly. Then each sample was vortexed for 30 seconds, followed by incubation at room temperature for 10 minutes and centrifugation at 2500 rpm for 5 minutes to obtain supernatant free of mucous and solid waste. The pellet was discarded, and the supernatant was taken in another set of respective microcentrifuge tubes, properly labeled according to each sample. Furthermore, 10% of fecal suspensions were stored at -20° C until the next analysis.

#### Enzyme immunoassay.

For detection of rotavirus VP6 antigen 10% fecal suspension was tested by using ProSpecTTM Rotavirus Microplate Assay kit (Cat No. 1185727, Oxoid Ltd., Basingstoke Hants, UK) as being used previously [23]. Briefly, 100µl of each diluted specimen, one positive control and one negative control were added to separate microwells. Positive and negative controls were provided in the kit. Then conjugate (100µl) was added to each microwell, followed by incubation at room temperature for 1 hr. After incubation, excess contents were aspirated and each well was washed with Wash buffer (400µl) five times. After the final wash, the micro titration plate was inverted and tapped on absorbent paper to completely remove traces of the wash buffer. Then 100µl of substrate was added to each well followed by incubation at room temperature for 10 minutes. The color changed immediately after incubation, it was an intense blue color indicating the presence of Group A rotavirus. While negative control remained colorless. The reaction was stopped by the addition of stop solution (100µl) in each well. The absorbance value for each sample was determined spectrophotometrically at 450nm on the plate reader. The cutoff value was calculated by adding 0.22 absorbance units to the negative control value, all samples were considered positive if their absorbance value was greater than the cutoff value and vice versa for negative samples.

#### RNA extraction.

RNA was extracted from processed samples. For this purpose, 10% diluted stool samples were thawed at room temperature, followed by centrifugation at 10,000 rpm for 5 minutes to remove debris and particulate contaminants. The clear supernatant was pipetted in new sets of 1.5ml microcentrifuge tubes, labeled respectively. QIAmp viral RNA mini kit (QIAGEN, Helden, Germany) was used for RNA extraction from 10% stool dilutions, and the protocol provided within the kit was followed.

#### Detection of rotavirus by rRT- PCR.

Real-time reverse transcriptase polymerase chain reaction was used for detection of human group A rotavirus through amplification of gene segment 9 (VP7). AgPath-ID^TM^ one-step RT-PCR kit was used for this purpose; all instructions were followed according to the protocol provided with the kit. The assay was carried out to detect the presence of rotaviruses from 60µl of extracted RNA. First of all, mix 1 was prepared by pipetting primers 1µl of each 1, 2, 3 and 5µl of dsRNA that was thawed at room temperature in a 1.5 ml microcentrifuge tubes, another tube was prepared by adding 12.5µl of 2X buffer, 1.33 µl of PCR water, 1.67µl of Detection Enhancer, 1µl of 25X enzyme mix (1X/25µl) and 0.5µl of Taqman Probe. Mix 1 was incubated for denaturation of dsRNA and primers at 97° C for 5 min, followed by the addition of mix 2 so that the total volume of the reaction was 25µl. Then samples were placed in a real-time thermal cycler, the initial reverse transcription step was carried out at 45° C for 10 min, the initial PCR activation step at 95° C for 10 min followed by 45 cycles of amplification (denaturation at 95° C for 15 min, annealing and extension at 60° C for 1 min) in a real-time instrument (ABI 7500 real-time PCR machine). On completion of the reaction, an amplification plot was obtained and cT values were calculated for each sample and negative controls. Results were analyzed by Prism 7700 Sequence Detector and calculated threshold cycle (Ct) values.

#### Genotyping of rotavirus.

**RT-PCR or round 1 PCR:**  Rotavirus gene segment 9 encoding VP7 protein was reverse transcribed into cDNA and 1062 bases long fragment of cDNA was generated. Similarly, 876 bp gene segment 4 that encodes VP4 protein was also reverse transcribed using QIAGEN^®^ One-Step RT-PCR Kit (cat. # 210210, Qiagen, Hilden, Germany) as instructed by the manufacturer. Reaction mix was prepared in 1.5 ml microcentrifuge tube by addition of 5 µl of QIAGEN buffer (5X) containing 2.5mM MgCl_2_, 1 µl of dNTPs mix (400µM of each dNTP), 12.25 µl of RNase free PCR water, 1.5 µl of respective primers (0.6 µM of each primer/reaction), 1µl of Enzyme mix and 0.25 µl of RNase inhibitor (5–10 units/reaction). The total volume of the reaction was 25 µl. The reaction was set up on ice to avoid denaturation of Enzymes. Consensus primers Beg9 and End9 (for VP7) and Con3 and Con2 (for VP4) were used for the amplification of respective gene fragments (S2 Table in S1 File), as described previously [24,25]. RNA (10ul) was added in respectively labeled PCR tubes, followed by dsRNA denaturation at 97° C for 5 min (BioRad Thermocycler). It was immediately placed on ice to maintain the effect of heat shock denaturation of dsRNA. Then 2.5µl of template RNA was added in respective labeled PCR reaction tubes already prepared with remaining reagents. All steps were carried out in a separate biosafety cabinet for PCR reaction to avoid chances of cross-contamination. The initial reverse transcriptase reaction for the VP7 gene was carried at 50° C for 30 min, followed by the initial PCR activation step at 95° C for 15 min (It activates HotStar Taq DNA Polymerase, inactivate reverse transcriptases, and denatures the cDNA template). It was followed by 40 cycles of amplification (denaturation: 94° C for 45 seconds, annealing: 42° C for 1 minute, extension: 72° C for 1 minute) and final extension at 72° C for 7 minutes in 9700 thermal cycler (Applied Biosystems). For the VP4 gene same thermal conditions were followed except primer annealing temperature, which was 50° C.

The 1^st^ round product was electrophoresed for analysis of PCR product on 1.5% agarose gel at 400 mA and 120 volts for 30–40 minutes.

**Semi-nested multiplex - PCR:**  During 2^nd^ round of PCR for G typing, VP7 reverse (End9) primer was used along with (G1-G4, G9, and G12) type specific primers as used in previous protocols [24,26]. For P typing, (con3) was used along with P[4], P[6], P[8], P[9], P[10] specific primers (S2 Table) as described in previously [27]. The reaction mix was prepared by pipetting 5 µl ABI-II 10X PCR Buffer (1X/50 µl), 4 µl of 25 mM MgCl_2_, 2 µl of 10 mM dNTPs, 29.5 µl of PCR water, 1 µl of each primer (10 µM), and 0.5 µl of Taq Polymerase (5U/µl) in 1.5 ml microcentrifuge tubes. It was prepared separately for G and P types by adding respective sets of primers of the above final concentration. Then 49µl of prepared mix from a total number of calculated reactions was added in respectively labeled PCR tubes by using micropipettes. PCR Tubes contained all the reagents except the cDNA template. Then 1µl of cDNA template from 1^st^ round RT-PCR was pipetted in respective tubes in separate PCR rooms to avoid possible contamination.

Then samples were placed in a thermal cycler, the thermal profile for the reaction was [28], initial PCR activation at 94° C for 2 min followed by 30 cycles of amplification (denaturation: 94° C for 1 min, annealing: 42° C for 1 min, extension: 72° C for 1 min) and final extension of DNA at 72° C for 7 min in veriti thermal cycler (Applied Biosystems). These thermal conditions were the same for both VP4 (P-type) and VP7 (G-type) genotyping. After the reaction was completed, the PCR products and negative controls were electrophoresed on 1.5% agarose gel (formed by dissolving 1.5g of agarose in 100µl of 1X TBE) stained with Ethidium Bromide, along with 50 bp ladder for comparison of amplicon size, at 120 volts and 400 mA for 50 min. For this purpose, 10µl of PCR product was homogenized with 4 µl of 6X DNA-loading dye (Fermantas) and was loaded on Gel along with 3 µl of DNA ladder. After Gel electrophoresis, the gel was analyzed using Molecular Imager Gel Doc XR with Quantity One software (BioRad). Gel doc pictures were taken and the gel was also analyzed under Trans UV illuminator to reevaluate reported types and mixed infections.

Genotypes were assigned to all processed samples based on the molecular size of PCR products. Each genotype had a specific amplicon size and was compared with 50 bp ladder to determine G and P types for all samples.

### Statistical analysis

Statistical analysis was carried out using SPSS version 20, and an online Chi-square calculator http://www.socscistatistics.com/tests/chisquare/default2.aspx. The level of confidence was set as 95% and a P ≤ 0.05 was considered as significant. The chi-square test was used to find the association of demographic and clinical features of disease with the rotavirus and its predominating genotypes. Mean ± standard deviation and percentages were calculated by MS Excel. Percentage = (no. of patients ÷ total no. of samples) × 100.

## Results

### Prevalence and RVA genotypes

**Fig 1:**
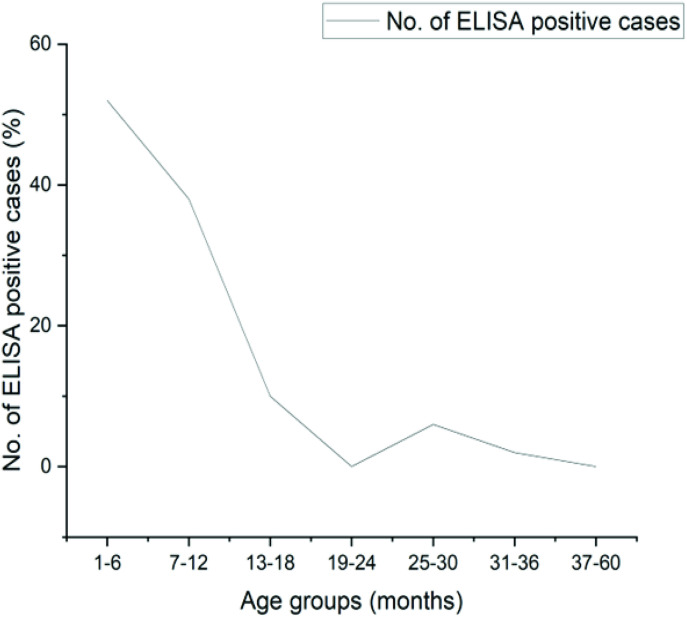
Distribution of RVA in children of various age groups during the study in 2014-2015 in Rawalpindi- Pakistan (mean± SD = 11.87 ± 16.75) months, age range 1-60 months.

Of the total 300 samples, 142 (47%) children were found positive for RVA VP6 inner capsid protein by ELISA. Out of 47%, 90% of children < 1 year of age were found infected with RVA (ϰ² 4.05, *P* = 0.044). RVA prevalence was highest (52%) in children 1–6 months of age (Fig 1). Males were more infected as compared to females (*P* = 0.405).

We detected 195 (65%) children positive for RVA through real-time RT-PCR. Subsequently, 159 (53%) samples were successfully genotyped for RVA through PCR (S1 Table in S1 File). Most common genotypes found in this study included G12P[6] (17.24%), G1P[8] (12.07%), G1P[4] (6.90%), G1P[6] (5.17%), G3P[6] (5.17%). Twenty-one percent of cases were found to be infected with mixed genotypes. The percentage prevalence of the RVA genotypes found during the study is listed below in Table 1.

**Table 1:** Distribution of VP7 and VP4 types of Rotavirus A in children with diarrhea.

Genotypes	No. of strains (%)
**G1**	82.8 (27.6)
G1P[8]	12.1
G1P[6]	5.17
G1P[4]	6.9
G1P[NT]	3.45
**G2**	15.5 (5.17)
*G2P[6]	3.45
G2P[4]	1.72
**G3**	36.3 (12.1)
*G3P[8]	1.72
G3P[6]	5.17
G3P[4]	3.45
G3P[NT]	1.72
**G4**	5.16 (1.72)
*G4P[6]	1.72
**G9**	16 (5.33)
G9P[6]	1.72
*G9P[4]	3.45
**G12**	14 (24.1)
G12P[8]	3.45
*G12P[6]	17.2
*G12P[4]	1.72
G12P[4] P[6]	1.72
**Mixed infection**	63 (21)
G2G3 P[4]	1.72
G1G12 P[6]	1.72
G2G12 P[6]	1.72
G1G3 P[8] or P[6]	3.45
G3G12 P[4] or P[6]	3.45
G1G9 P[4] or P[6]	3.45
G1G3G9 P[6]	1.72
GNT or G12 P[4]P[6]	3.45
**GNT P[6]**	10.3 (3.43)
**Total (%)**	158 (52.7)

* depicts unusual genotype combination (not detected frequently).

NT: non-typeable strains

### Clinical features of RVA gastroenteritis/RVA strains

The mean duration of illness of patients before discharge was (4.67 ± 2.93 days). RVA-positive (42%) cases were significantly associated with fever, and a high number (84.6%) of patients were found with short duration (1–3 days) of diarrhea (Table 2). Out of 83 children found with vomiting, 47% of non-RVA diarrheal infants were significantly associated with 1–6 episodes of vomiting than 39.7% of RVA-infected patients (*P* < 0.05) (Table 2).

**Table 2:** Association of clinical features with rotavirus and non-rotavirus gastroenteritis.

Clinical features	Rotavirus GE patients n = 142 (47%)	Non-rotavirus GE patients n = 158 (53%)	*P*- value
Fever (>37.5 °C)			(χ2 = 5.002) P = 0.025
Yes	60 (42.3)	35 (22.4)	
No	82 (57.6)	123 (77.6)	
Vomiting			(χ2 = 1.652) P = 0.438
Yes	112 (78.8)	114 (72.4)	
No	31 (21.2)	44 (27.6)	
Duration of diarrhea (days)			(χ2 = 7.97) P = 0.004
1-3	120 (84.6)	95 (60.3)	
4-6	22 (16)	63 (39.6)	
Duration of vomiting (days) 1-3 days 4-6 days	90 (63.4) 22 (16)	74 (47) 41 (26)	(χ2 = 2.72) P = 0.09
VE/24 hour			(χ2 = 4.1) P = 0.04
1-6	23 (63.5)	106 (67.3)	
7-12	22 (15.4)	5 (3.2)	
15	0	3 (2)	
DE/24 hour			(χ2 = 1.21) P = 0.27
8-15	85 (59.8)	112 (70.7)	
16-23	46 (32.7)	38 (24.1)	
24-31	11 (7.7)	5.5 (3.45)	
60	0	2.7 (1.72)	
Median duration of hospitalization	4	5	

VE: vomiting episodes, DE: Diarrheal episodes

We related the three predominant genotypes (G12P[6], G1P[8], G1P[4]) in our study with clinical symptoms of RVA infection, hence, G12P[6] was found associated with fever significantly (Table 3), while no genotype had any association with diarrheal episodes (*P* > 0.05). The individual genotypes and mixed infections (88%) were specifically more common in infants less than 1 years of age.

**Table 3:** Distribution of RVA genotype combination by child’s age and clinical symptoms.

Clinical symptoms		Common rotavirus genotype combinations (%)		
**G1P[8]**	**G12P[6]**	**G1P[4]**
**7**	**10**	**4**
**Fever**				
**(Age)**	**<1 year**	**28.5**	**80**	**0**
	**>1 year**	**0**	**0**	**0**
	**Total**	**28.5**	80*	**0**
**Vomiting**	**<1 year**	**51**	**80**	**50**
	**>1 year**	**20**	**0**	**25**
	**Total**	**71**	**80** **ǂ**	**75**
**RDE/ day**	**8-15**	**4.5**	**5.4**	**2.7**
	**16-23**	**0.9**	**2.7**	**0.9**
	**24-31**	**0.9**	**0.9**	**0**
	**60**	**0**	**0**	**0**
**VE/ day**	**1-6**	**60**	**88**	**100**
	**7-12**	**40**	**12**	**0**

*G12P[6] diarrheal episodes were more associated with fever as compared to G1P[8] (χ2 4.496, *P* = 0.03) ǂG12P[6] was associated with highest vomiting episodes, but not significantly (*P* > 0.05).

## Discussion

Despite the introduction of the rotavirus vaccine a decade ago, RVA is still a major cause of diarrhea and dehydration in young children, worldwide [12,29]. In this study, children < 5 years of age, admitted to RGH during 2014–2015 and presented with the symptoms of gastroenteritis, were studied for rotavirus A presence. We report a fair rise in RVA gastroenteritis cases in the region, with the highest prevalence (47% by ELISA) of rotavirus, which is quite higher than the prevalence (23.8%) rate in the same region of Rawalpindi, during 2010, and even higher than 26.8% during 2016 [3,28]. The rise in RVA infection agrees with the other hospital-based studies conducted in some countries of Africa and Asia [29–32], and in Eastern Mediterranean Regions [33], with the rate of RVA prevalence between 40–50% and 40%, respectively. Moreover, the screening of stool samples by real-time PCR resulted in a 65% prevalence of RVA and 53% by conventional (c RT-PCR). Indeed, the difference in RVA detection between the three molecular techniques in this study is based on the differential sensitivity of these techniques.

Overall, in the present study, 90% of the infected infantile population was less than one year of age, which could be due to a low degree of passive immunity conferred by maternal antibodies following the initial months of life [34]. Such findings reinforce the previous reports from Rawalpindi [28], Faisalabad [35] and Karachi [18], with the greater susceptibility of children < 1 year of age towards RVA gastroenteritis and associated mortality.

RVA infection is mostly associated with fever, vomiting, and watery diarrhea [36], however, its clinical symptoms specifically relevant to acute gastroenteritis caused by rotavirus need investigation, to avoid unnecessary use of antibiotics. Apart from the frequent vomiting and watery diarrheal episodes, we found fever as the most significant indicator of RVA infection (Tables 2 and 3). Similarly, in concordance with a previous systematic hospital-based surveillance study in Pakistan, we report that non-RVA gastroenteritis patients suffered a significantly long duration of diarrhea and frequent vomiting per day as compared to the patients with RVA gastroenteritis (Table 2). Furthermore, a significant number of patients suffering from fever were found infected with G12P[6], than those patients infected with either G1P[8] or G1P[4] strain. Such an association suggests the possibility of a low level of passive immunity against this newly emerging G12P[6] strain during 2014–2015.

We report a unique changing trend of prevalent genetic combinations, as unlike the previously dominating strain G1P[8] [28] we detected G12P[6] as a dominant (17.2%) genotype in Rawalpindi, followed by G1P[8] and G1P[4]. G12P[6] had been found at a very low percentage (11%) in Bangladesh [35,37,38] and 2.6%-7% in other countries of Asia and Africa [17,29,32,38–40]. Within Pakistan, G12P[6] was identified with a prevalence of 4.04% by Kazi *et al* during 2006–2008 and 6.7% by Tamim *et al* during 2010, however, its specificity with P[4] had not been reported previously as found in the present study [20,23,35]. In the studies conducted after 2010, G12 in combination with P[6] and P[8] has started as emerging genotypes in the world [2,41]. However, our study is the first report on the significantly high prevalence of G12 P[6] in Rawalpindi-Pakistan. The G12 genotype was first identified in the Philippines in 1987 but remained relatively rare until the early 2000s [42]. Since then, G12 strains, particularly G12P[6] and G12P[8], have been increasingly reported in Asia, Africa, Europe, and the Americas [21,43,44]. G12P[6] is often associated with reassortment events, where genetic material is exchanged between different rotavirus strains, leading to new combinations [45]. G12P[6] has been frequently detected in countries like India [46], Bangladesh [47], and Nepal [48]. In some regions, it has become one of the dominant strains. Reports of G12P[6] have been documented in countries such as Malawi, Kenya, and South Africa. While less common G12P[6] has also been identified in Europe and the Americas, [49] is often linked to travel or importation. Pakistan introduced the rotavirus vaccine (Rotarix, G1P[8]) into its Expanded Program on Immunization (EPI) in 2017 [12]. The vaccine has shown effectiveness in reducing the burden of rotavirus disease, but its impact on less common strains like G12P[6] is still being studied [50]. G12P[6] has been identified in Pakistan as part of rotavirus surveillance studies, particularly in the last decade [51]. It is one of the less common genotypes compared to dominant strains like G1P[8] and G9P[8], but its detection highlights the ongoing genetic diversity of rotavirus in the region.

We detected some genotype combinations for the first time in Pakistan, which include G3P[4], G3P[6], and G12P[4]. Other unusual types found during our study were G3P[8], G9P[4], G4P[6], and G2P[6] which have a probable origin from animals [52]. In the same way, mixed-type infection fairly raised (21%) in our study in converse to its previous (6.7%) prevalence [28] in Rawalpindi. However, it is consistent with a similar trend in other developing countries of Asia [53,54]. As the G3 RVA strain has been identified in only one study in the past, with only P[8] specificity in Faisalabad, Pakistan [35], we report the G3 type as the third prevalent (12%) type for the first time in the current epidemiological area of study with the P[8], P[4] and P[6] types. As G3 and G4 were absent previously in the same zone [20,28], its current upsurge confirms the changing pattern of predominant genotypes as found in Bangladesh [55], China [56], and in other regions of Pakistan [20,23,35,57]. Moreover, G3 and G4 have been previously reported as declining in the Indian subcontinent with the rise of G9 and G12 genotypes [58].

Twelve percent of samples were found either G or P non-typeable, which could be due to mutants or unidentified strains. High cases of mixed infections are a source of unusual or mutant strains that arise by either point mutation or genetic reassortment among co-infecting strains [59].

G12P[6] along with the most genotypes found during our study were prevalent in children < 1 year of age. Moreover some genotypes (G12P[6], G1P[8] and G1P[4]) were only prevalent in males and vice versa. Such gender specific RVA distribution could be due to the greater susceptibility of respective gender towards that specific genotype or they may have some genetic relationship on immunological basis that should be investigated in detail.

Pakistan is among developing countries facing multiple health and economic challenges, out of which, viral infections pose a major health risk, especially rotavirus is causing a significant disease burden in the infantile population. Pakistan introduced the Rotarix vaccine in 2018; such intervention in challenging conditions of a country where the vaccine already proved less than 30% effective, requires an updated estimate of RVA disease burden, particularly the predominant genotypes, circulating in pre-vaccine time, in the region. The genotypes exhibit temporal and spatial variations, thus, one type prevalent at any one time may sharply decline at another time [60] [61] as our study reported the first upsurge of G12P[6], thus emphasizing on surveillance of rotavirus and genotypic analysis at national level.

The current report on the rapid emergence and adaptation of G12P[6] along with the rise of previously declining G3 serotypes or unusual strains in Rawalpindi, Pakistan, would provide the first upsurge of G12P[6] in the zone, along with its influence on the effectiveness of rotavirus vaccine introduced in resource-limited settings of Pakistan. The high rotavirus disease burden and the emergence of G12P[6] and other rare genotypes highlight the need for a multi-faceted public health response. This includes strengthening surveillance, ensuring high vaccine coverage, and investing in research to address evolving rotavirus strain diversity. These efforts are critical to reducing the global burden of rotavirus disease and achieving equitable child health outcomes.

## Conclusion

Rotavirus gastroenteritis is a significant health burden in Pakistan associated with obvious clinical symptoms in 2015. The clinical symptoms cause significant health burdens and malnutrition among growing kids in Pakistan. We provide the baseline data on RVA prevalence and unique genotypic profile in 2014–2015. G12P[6] is an emerging rotavirus genotype with a growing presence in multiple regions. Its detection highlights the dynamic nature of rotavirus epidemiology and the importance of sustained vaccination and surveillance efforts. G12P[6] is associated with moderate to severe diarrhea in children, similar to other common rotavirus strains like G1P[8] and G9P[8]. Its emergence highlights the importance of ongoing surveillance to monitor rotavirus evolution and the effectiveness of vaccines. Rotavirus vaccines, such as Rotarix (monovalent, G1P[8]) and RotaTeq (pentavalent, G1-G4, P[8]), have significantly reduced the burden of rotavirus disease globally. However, the emergence of strains like G12P[6] raises questions about potential vaccine escape and the need for broader protection in future vaccine formulations. The spread of G12P[6] underscores the need for continued rotavirus surveillance to track strain diversity and inform vaccine development. Efforts to improve vaccine coverage, especially in low- and middle-income countries like Pakistan, remain critical to reducing the global burden of rotavirus disease.

## Supporting information

S1 File Refer to supporting information file for Table S1-Table S5 and Figure S1- Figure S5. (ZIP)
