# Supplementary material for: Epidemiology and clinical features of Rotavirus infection among children in Rawalpindi, Pakistan
Source: PLoS One. 2025 May 20;20(5):e0324037. doi: 10.1371/journal.pone.0324037 (PMC12091768; doi:10.1371/journal.pone.0324037)
Supplement: S1 File — (ZIP) [file pone.0324037.s001.zip › supporting information PLOS rotavirus/S1_table Pdf.pdf]

Supporting Information

Table S1. Prevalence of group A rotavirus by ELISA, Conventional PCR (cPCR) and real-time PCR (rRT-PCR) assay, the difference in detection rates was based on the sensitivity of three molecular-based tests

Rotavirus	ELISA	cPCR	rRT-PCR
Positive	142 (47%)	159 (53%)	195 (65%)
Negative	158 (53%)	141(47%)	105 (35%)
Total			300
