# Supplementary material for: Epidemiology and clinical features of Rotavirus infection among children in Rawalpindi, Pakistan
Source: PLoS One. 2025 May 20;20(5):e0324037. doi: 10.1371/journal.pone.0324037 (PMC12091768; doi:10.1371/journal.pone.0324037)
Supplement: S1 File — (ZIP) [file pone.0324037.s001.zip › supporting information PLOS rotavirus/S2_fig.pdf]

Supporting Information

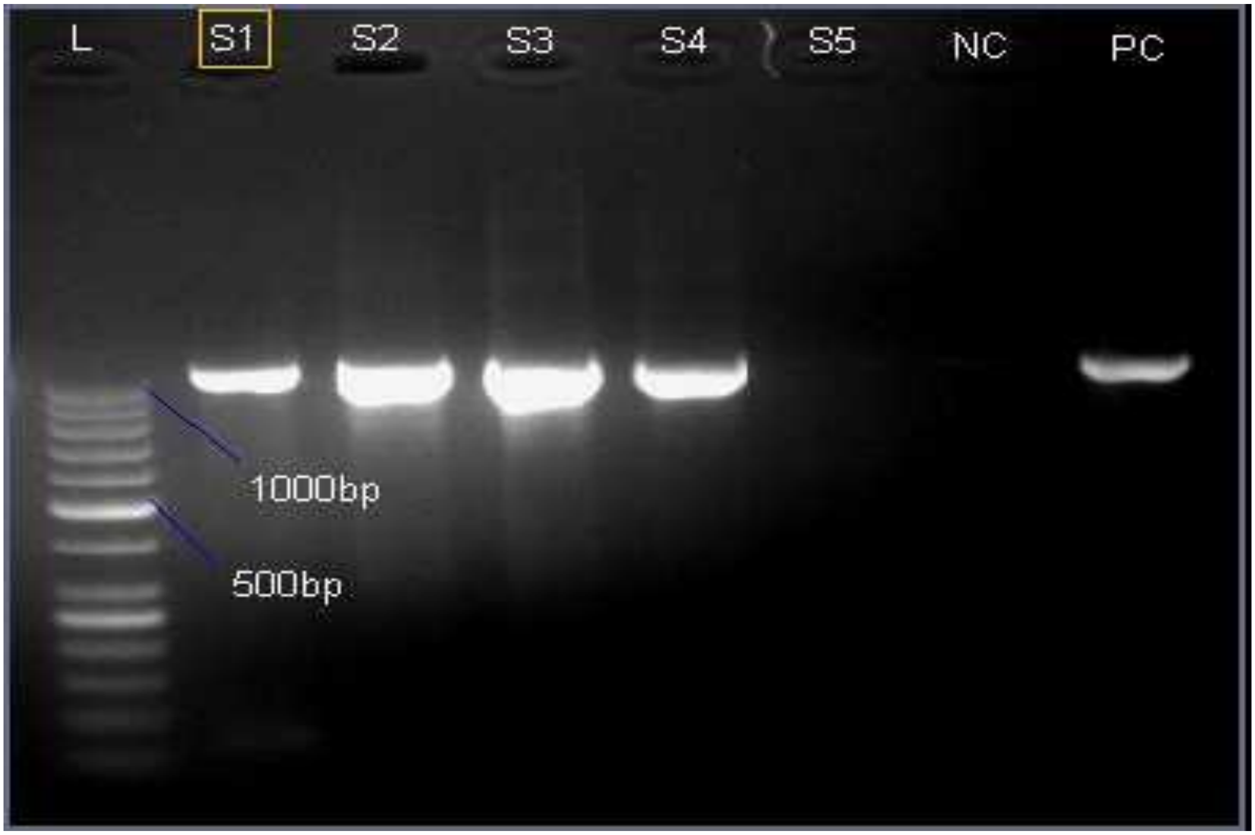

Figure S2. Gel results showing a successfully amplified product of RT-PCR**.** In this case, the targeted region of amplification was 1062 base pair gene fragment VP7 for G typing. The first well contained Ladder (L) of 50 bp, followed by five samples (S1-S5), a negative control (NC), and a positive control (PC). The first four samples were positive for G type, except sample 5.
