# Supplementary material for: Epidemiology and clinical features of Rotavirus infection among children in Rawalpindi, Pakistan
Source: PLoS One. 2025 May 20;20(5):e0324037. doi: 10.1371/journal.pone.0324037 (PMC12091768; doi:10.1371/journal.pone.0324037)
Supplement: S1 File — (ZIP) [file pone.0324037.s001.zip › supporting information PLOS rotavirus/S2_table.pdf]

Supporting Information

Table S2. Sequences of Primers used for genotyping of rotavirus based on VP-7 and VP-4 Genes

**Gene**	**Primer**	**Primer Sequence**	**Nucleotide position**	**Amplicon size (bp)**	**Genotype**
VP-7 Gene	Beg 9	GGC TTT AAA AGA GAG AAT TTC CGT CTG G	1-28	1062	
	End 9	GGT CAC ATC ATA CAA TTC TAA TCT AAG	1062-1036		
	VP7F	GGC TTT AAA ARM GAG AAT TTC CG	1-23	Generic Forward Primer	
	G1-R	ACA TTA GAR CCA CCA ACT TGT AT	850-828	849	G1
	G2-R	CAT TAT AAT CAC AAT ACA GTT G	147-127	146	G2
	G3-R	CGT CAG TAA TYA CTA RTT TYT CAG CTG	733-707	732	G3
	G4-R	GAG CAT TCG MTA ATA MTG ATA ATA C	175-199	198	G4
	G9-R	CAG AGT ATY YTT CCA TTC HGT ATC TCC	328-354	353	G9
	G12-R	GAA GTC ATA AAA YTY TCT TGT TG	262-284	283	G12
VP-4 Gene	Con 3	TGG CTT CGC CAT TTL ATA GAC A	11-32	876	Consensus primer
	Con 2	ATT TCG GAC CAT TTA TAA CC	887-868		
	VP4F	TGG YTT CVC TCA TTT ATA GAC A	11-32	Generic Forward Primer	
	P[4]-R	GCA TYC CTA CAA GTC TAT TAY TAG	508-485	497	P[4]
	P[6]-R	ACC ATC GAG TAC TGG YTC TAT YGT TG	210-185	199	P[6]
	P[8]-R	GYG GTT CAA YAG CAA CKA CT	350-330	339	P[8]
	P[9]-R	TGA GAC ATG CAA TTG GAC	402-385	391	P[9]
	P[10]-R	ATC ATA GTT AGT AGT CGG	594-575	583	P[10]

F= forward; R= reverse; VP= viral structural protein; bp= base pair; R= A or G; M= A or C; Y= C or T; H= A, C or T.
