# Supplementary material for: Epidemiology and clinical features of Rotavirus infection among children in Rawalpindi, Pakistan
Source: PLoS One. 2025 May 20;20(5):e0324037. doi: 10.1371/journal.pone.0324037 (PMC12091768; doi:10.1371/journal.pone.0324037)
Supplement: S1 File — (ZIP) [file pone.0324037.s001.zip › supporting information PLOS rotavirus/S3_fig.pdf]

# Supporting Information

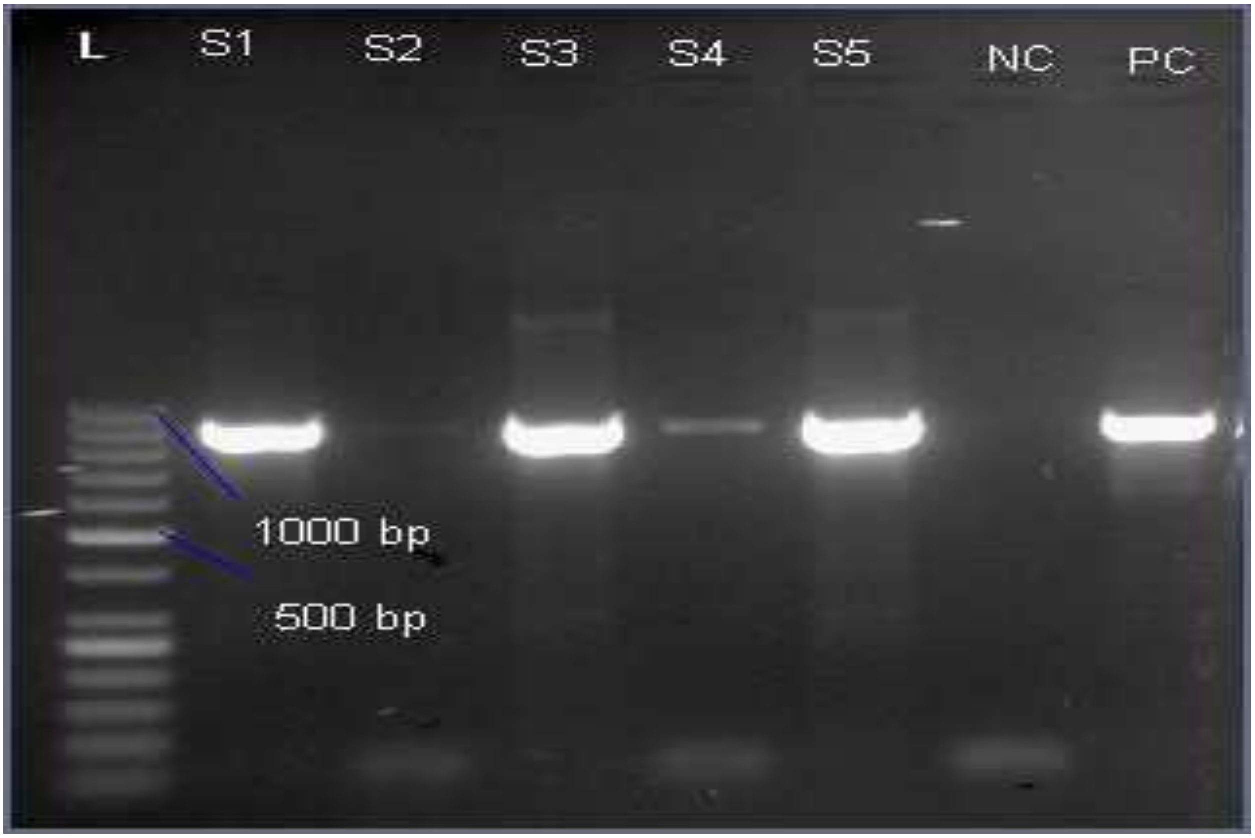


**Figure S3.** Gel results show a successfully amplified VP4 gene segment by round one PCR. The targeted gene segment belonged to gene segment 4, containing 876 bp. The first well contained a Ladder (L) followed by five samples (S1-S5), a negative control (NC), and a Positive control (PC).
