# Supplementary material for: Epidemiology and clinical features of Rotavirus infection among children in Rawalpindi, Pakistan
Source: PLoS One. 2025 May 20;20(5):e0324037. doi: 10.1371/journal.pone.0324037 (PMC12091768; doi:10.1371/journal.pone.0324037)
Supplement: S1 File — (ZIP) [file pone.0324037.s001.zip › supporting information PLOS rotavirus/S3_table.pdf]

Supporting Information

Table S3. Association of various anonymous age groups with mean diarrheal and vomiting episodes/24 hour during 2014-2015

Groups	Mean diarrhea episodes/24h	Mean vomiting episode/24h
1	15.2 (8-60)	3.2 (1-10)
2	15.19 (10-25)	3.85 (1-15)
3	13.4 (10-16)	3.6 (2-6)
4	15.67 (15-16)	4 (4)
5	14.4 (10-20)	5.67 (3-9)
6	10 (10)	N
7	13.4 (10-20)	2.67 (1-4)
Total	14.96 (10-60)	13.6 (0-15)

N = no
