# Supplementary material for: Epidemiology and clinical features of Rotavirus infection among children in Rawalpindi, Pakistan
Source: PLoS One. 2025 May 20;20(5):e0324037. doi: 10.1371/journal.pone.0324037 (PMC12091768; doi:10.1371/journal.pone.0324037)
Supplement: S1 File — (ZIP) [file pone.0324037.s001.zip › supporting information PLOS rotavirus/S4_fig.pdf]

Supporting Information

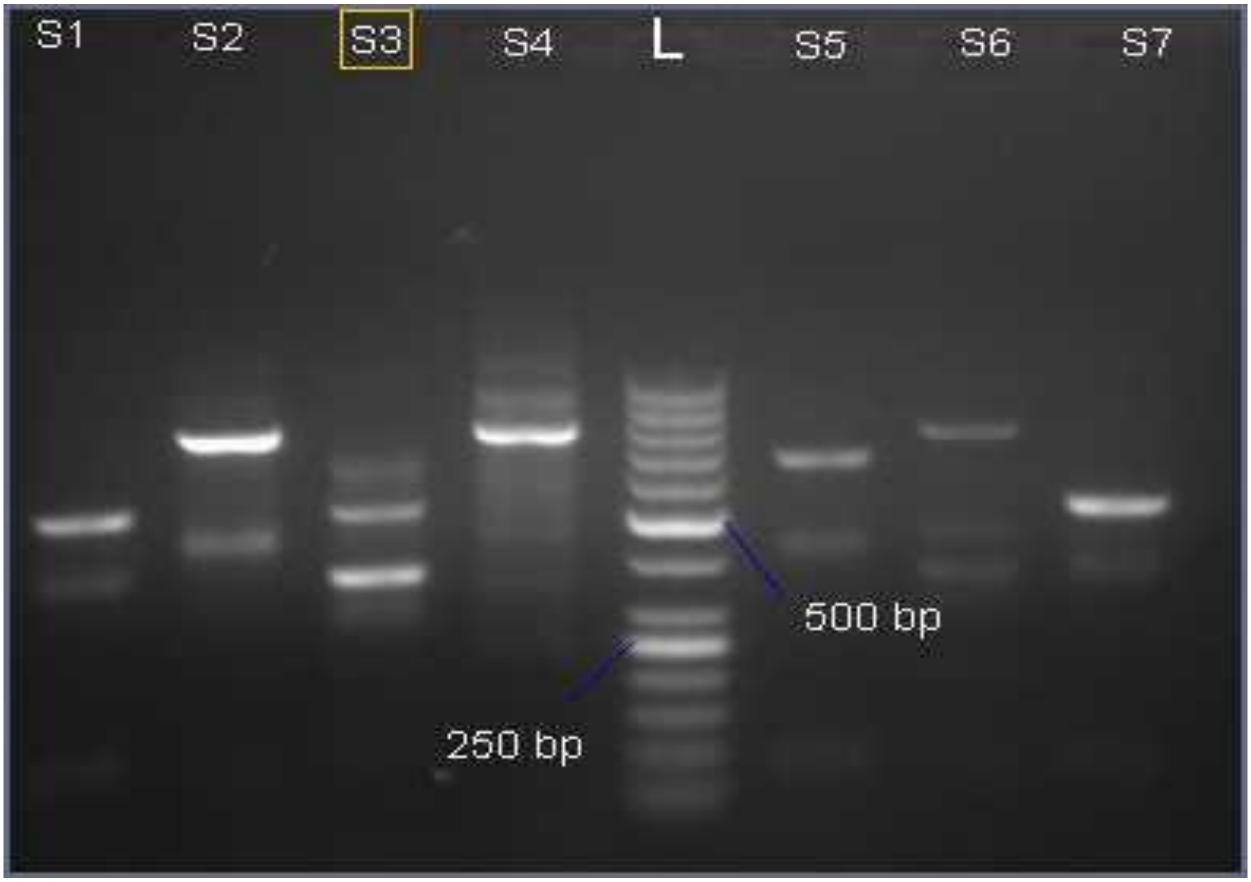

Figure S4. Gel results showing successfully amplified gene segments 9 for G typing in round 2 PCR**.** The first sample was G12 (526 bp), sample 2, sample 4, and sample 6 were G1 (749 bp), samples 3 and 7 were G12. A ladder (L) of 50 bp was run in the middle to compare the size of segments.
