# Supplementary material for: Epidemiology and clinical features of Rotavirus infection among children in Rawalpindi, Pakistan
Source: PLoS One. 2025 May 20;20(5):e0324037. doi: 10.1371/journal.pone.0324037 (PMC12091768; doi:10.1371/journal.pone.0324037)
Supplement: S1 File — (ZIP) [file pone.0324037.s001.zip › supporting information PLOS rotavirus/S4_table.pdf]

Supporting Information

Table S4. Association of duration of illness with the children of various age group categories

Groups	Mean illness duration (days)	Mean diarrhea duration (days)	Mean vomiting duration (days)
1	4.22 (1-11)	2.94 (1-6)	3 (1-6)
2	4.86 (1-17)	3 (1-6)	3 (1-6)
3	6.6 (3-11)	2.8 (1-5)	2.8 (1-5)
4	4 (2-7)	3 (2-4)	2 (2)
5	5 (2-8)	4.6 (2-6)	4 (2-6)
6	13 (13)	3 (3)	N
7	4.8 (1-9)	3 (2-4)	3.33 (1-6)
