# Supplementary material for: Epidemiology and clinical features of Rotavirus infection among children in Rawalpindi, Pakistan
Source: PLoS One. 2025 May 20;20(5):e0324037. doi: 10.1371/journal.pone.0324037 (PMC12091768; doi:10.1371/journal.pone.0324037)
Supplement: S1 File — (ZIP) [file pone.0324037.s001.zip › supporting information PLOS rotavirus/S5_fig.pdf]

Supporting Information

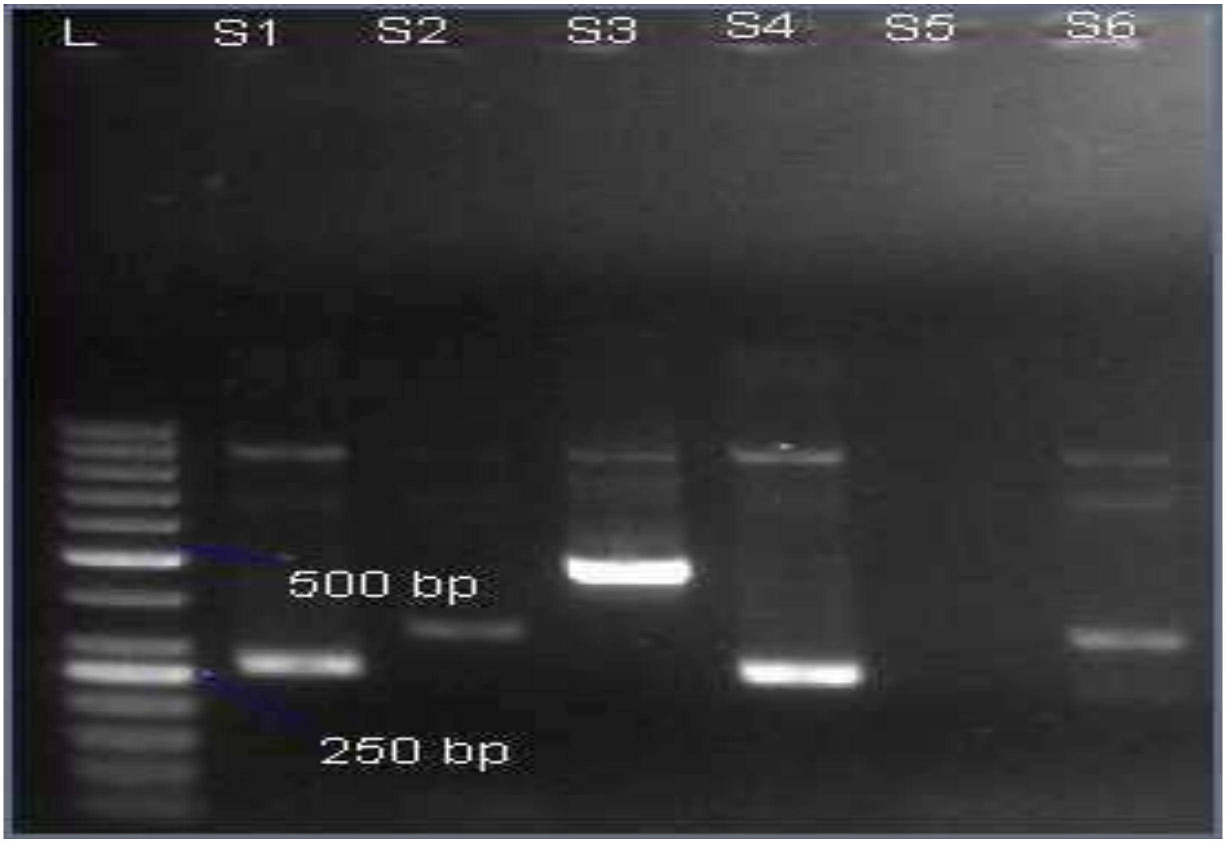

Figure S5. Gel results of round 2 PCR for gene segment 4 of rotavirus**.** The first well contained a Ladder (L) of 50 bp. The second well shows P[6] type of 267 bp size. The sample two contained P[8] type of 345 bp, Sample 3 with P[4] type of 483 bp, sample 4 and sample 6 with P[6] type, and sample 5 was negative for any P type.
