# Supplementary material for: Epidemiology and clinical features of Rotavirus infection among children in Rawalpindi, Pakistan
Source: PLoS One. 2025 May 20;20(5):e0324037. doi: 10.1371/journal.pone.0324037 (PMC12091768; doi:10.1371/journal.pone.0324037)
Supplement: S1 File — (ZIP) [file pone.0324037.s001.zip › supporting information PLOS rotavirus/S5_table.pdf]

Supporting Information

Table S5. Collection of stool samples from various studied age groups (Anonymous)

Group categories	Number of collected samples
1	150
2	98
3	14
4	8
5	14
6	2
7	14
Total	300
